# Supplementary material for: The causal role of circulating inflammatory markers in osteoporosis: a bidirectional Mendelian randomized study
Source: Front Immunol. 2024 Jul 18;15:1412298. doi: 10.3389/fimmu.2024.1412298 (PMC11291241; doi:10.3389/fimmu.2024.1412298)
Supplement: Supplementary file 1 [file DataSheet_1.zip › supplementary material/OSTEOPOROSIS-CIM/GCST90274780.tsv.gz_forest.pdf]

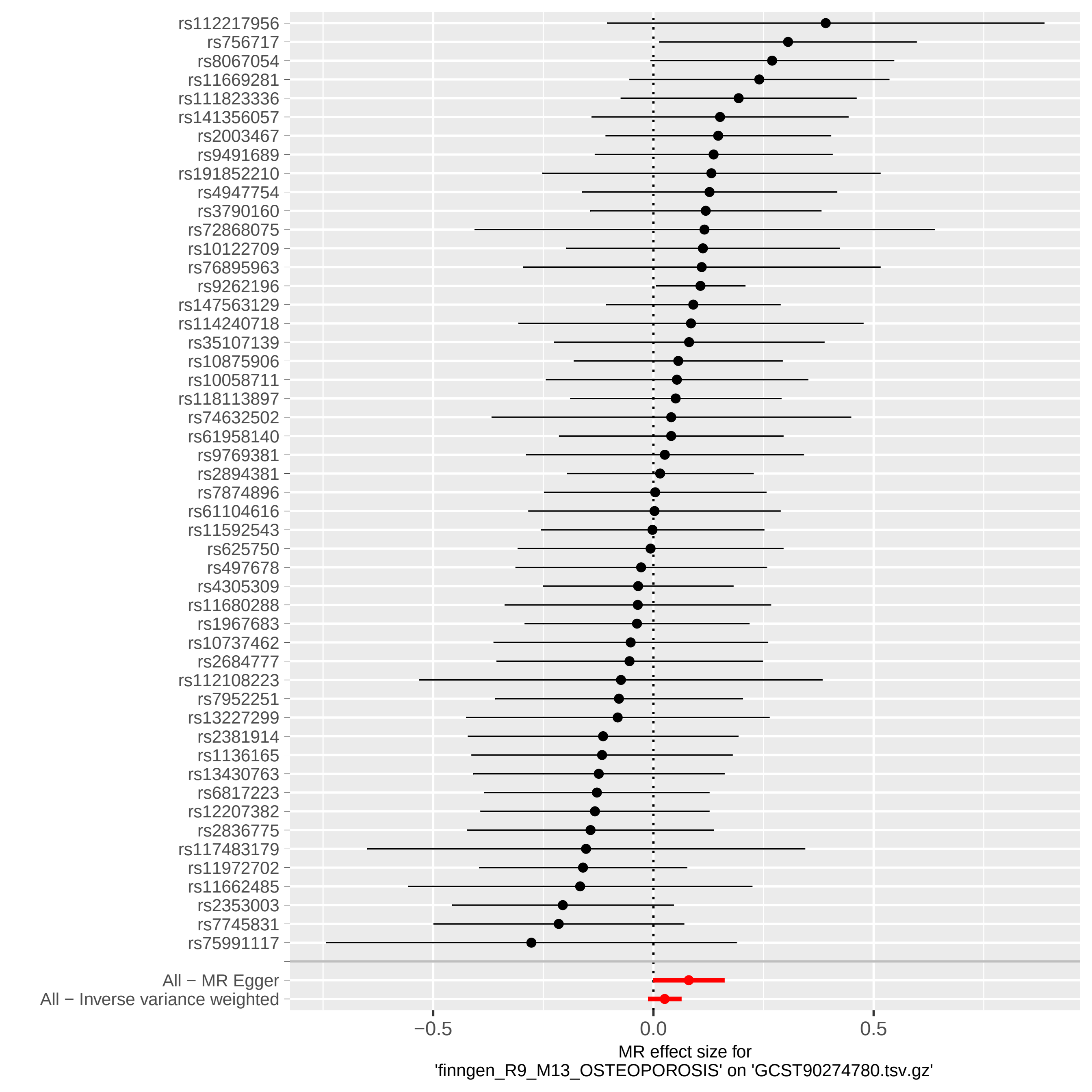
rs112217956
rs756717
rs8067054
rs11669281
rs111823336
rs141356057
rs2003467
rs9491689
rs191852210
rs4947754
rs3790160
rs72868075
rs10122709
rs76895963
rs9262196
rs147563129
rs114240718
rs35107139
rs10875906
rs10058711
rs118113897
rs74632502
rs61958140
rs9769381
rs2894381
rs7874896
rs61104616
rs11592543
rs625750
rs497678
rs4305309
rs11680288
rs1967683
rs10737462
rs2684777
rs112108223
rs7952251
rs13227299
rs2381914
rs1136165
rs13430763
rs6817223
rs12207382
rs2836775
rs117483179
rs11972702
rs11662485
rs2353003
rs7745831
rs75991117
All − MR Egger
All − Inverse variance weighted
−0.5
0.0
0.5
MR effect size for
'finngen_R9_M13_OSTEOPOROSIS' on 'GCST90274780.tsv.gz'
